# Comment on Weitzman et al. Resistance to Antimalarial Monotherapy Is Cyclic. *J. Clin. Med.* 2022, *11*, 781

**DOI:** 10.3390/jcm11102934

**Published:** 2022-05-23

**Authors:** Rob W. van der Pluijm, Benjamin J. Visser, Arjen M. Dondorp

**Affiliations:** 1 Department of Internal Medicine & Infectious Diseases, Amsterdam University Medical Centers, University of Amsterdam, 1105 AZ Amsterdam, The Netherlands; b.j.visser@amsterdamumc.nl; 2 Mahidol-Oxford Tropical Medicine Research Unit, Faculty of Tropical Medicine, Mahidol University, Bangkok 10400, Thailand; arjen@tropmedres.ac; 3 Centre for Tropical Medicine and Global Health, Nuffield Department of Medicine, University of Oxford, Oxford OX3 7LG, UK

Weitzman et al. used PubMed text mining to determine the trends of antimalarial resistance over the last 40 years [1]. In short, the authors divided the number of citations each year mentioning ‘drug’ AND ‘malaria’ AND ‘resistance’ by the number of citations mentioning ‘drug’ OR ‘malaria’ OR ‘resistance’, using the names of different antimalarials in the ‘drug’ position of this formula. The resulting ‘normalised citations’ were then used as an indicator of resistance to the drug in that year. According to the authors, these normalised citations display cyclic patterns. This, according to the authors, indicates that ‘Resistance to antimalarial monotherapy is cyclic’.

We doubt whether this methodology is suitable to determine temporal patterns of antimalarial resistance. Firstly, we think the nominator in this formula could be ‘polluted’ by publications that happen to mention the drug of interest as well as the word resistance.

Secondly, the authors seemed to ignore other reasons for the increase or decline in normalised citations for specific antimalarials. Halofantrine, for example, was first marketed in 1984 [2]. Not surprisingly, the citation rate determined by Weitzman et al. increases after this year. The decline around 1995 is most likely not an effect of a sudden reduction in resistance, but rather the result of a strong reduction in the use of halofantrine due to concerns over the cardiotoxic side effects of halofantrine, which were first described around 1993 [3,4].

The (over)reliance on the methodology used by the authors leads to some confusing conclusions. The authors describe a linear rise in resistance for artemisinin, dihydroartemisinin and artemether up to 2020, yet a ‘plunge’ in artesunate resistance after 2005. Although the authors seemed to acknowledge that dihydroartemisinin is the active metabolite of artemisinin, artemether and artesunate, they suggest that ‘given the right conditions’, resistance to artemisinin and dihydroartemisinin could also ‘plunge’ from their linear rise in resistance, similar to that of artesunate. The authors even go as far as to state that the fact that only a ‘plunge’ is seen for artesunate ‘suggests more than one mechanism of action, unlike that of the artemisinins’. It is puzzling to understand why resistance to artesunate would have ‘plummeted’ since 2005, as resistance to artemisinins was first described only in around 2008–2009 [5,6]!

It is likely that resistance to antimalarials will follow a cyclic pattern as resistance could lead to cessation of the use of the antimalarial which will reduce drug pressure, which subsequently could result in a reduction in antimalarial resistance, provided that the resistant genotype is associated with a fitness cost in the absence of drug pressure. Similarly, chloroquine resistance has diminished after the cessation of chloroquine monotherapy in sub-Saharan Africa [7,8]. However, we think the methods used by Weitzman et al. are at great risk of drawing incorrect conclusions. In addition, we think the cyclic pattern of normalised citation rates of antimalarial resistance described by the authors cannot be used to predict the future prevalence of antimalarial resistance.
